# SARS-CoV-2 Induces Lymphocytopenia by Promoting Inflammation and Decimates Secondary Lymphoid Organs

**DOI:** 10.3389/fimmu.2021.661052

**Published:** 2021-04-28

**Authors:** Qun Xiang, Zeqing Feng, Bo Diao, Chao Tu, Qinghua Qiao, Han Yang, Yi Zhang, Gang Wang, Huiming Wang, Chenhui Wang, Liang Liu, Changsong Wang, Longding Liu, Rong Chen, Yuzhang Wu, Yongwen Chen

**Affiliations:** ^1^ Institute of Immunology, PLA, Third Military Medical University, Chongqing, China; ^2^ Department of Medical Laboratory Center, General Hospital of Central Theater Command, Wuhan, China; ^3^ Department of Pathology, Jinyintan Hospital, Wuhan, China; ^4^ Pingdingshan Medical District, The 989th Hospital of the PLA Joint Logistic Support Force, Pingdingshan, China; ^5^ Department of Nephrology, Renmin Hospital of Wuhan University, Wuhan, China; ^6^ Department of Forensic Medicine, Tongji Medical College, Huazhong University of Science and Technology, Wuhan, China; ^7^ Department of Pathology, 989^th^ Hospital of PLA, Luoyang, China; ^8^ Yunnan Key Laboratory of Vaccine Research and Development on Severe Infectious Diseases, Institute of Medical Biology, Chinese Academy of Medical Science and Peking Union Medical College, Kunming, China

**Keywords:** SARS-CoV-2, COVID-19, macrophages, dendritic cells, lymphocytopenia, spleen, lymph nodes

## Abstract

While lymphocytopenia is a common characteristic of coronavirus disease 2019 (COVID-19), the mechanisms responsible for this lymphocyte depletion are unclear. Here, we retrospectively reviewed the clinical and immunological data from 18 fatal COVID-19 cases, results showed that these patients had severe lymphocytopenia, together with high serum levels of inflammatory cytokines (IL-6, IL-8 and IL-10), and elevation of many other mediators in routine laboratory tests, including C-reactive protein, lactate dehydrogenase, α-hydroxybutyrate dehydrogenase and natriuretic peptide type B. The spleens and hilar lymph nodes (LNs) from six additional COVID-19 patients with post-mortem examinations were also collected, histopathologic detection showed that both organs manifested severe tissue damage and lymphocyte apoptosis in these six cases. *In situ* hybridization assays illustrated that SARS-CoV-2 viral RNA accumulates in these tissues, and transmission electronic microscopy confirmed that coronavirus-like particles were visible in the LNs. SARS-CoV-2 Spike and Nucleocapsid protein (NP) accumulated in the spleens and LNs, and the NP antigen restricted in angiotensin-converting enzyme 2 (ACE2) positive macrophages and dendritic cells (DCs). Furthermore, SARS-CoV-2 triggered the transcription of *Il6*, *Il8* and *Il1b* genes in infected primary macrophages and DCs *in vitro*, and SARS-CoV-2-NP^+^ macrophages and DCs also manifested high levels of IL-6 and IL-1β, which might directly decimate human spleens and LNs and subsequently lead to lymphocytopenia *in vivo*. Collectively, these results demonstrated that SARS-CoV-2 induced lymphocytopenia by promoting systemic inflammation and direct neutralization in human spleen and LNs.

## Introduction

In December 2019, clusters of patients with pneumonia of unknown etiology were reported by local health facilities in Wuhan, Hubei Province, China. Later, the causative agent responsible for this mysterious pneumonia was officially named severe acute respiratory syndrome coronavirus 2 (SARS-CoV-2) by the International Committee on the Taxonomy of Viruses, and the disease it caused was designated as coronavirus disease 2019 (COVID-19) (1). According to the daily reports of the WHO, the SARS-CoV-2 pandemic has caused 102,458 laboratory confirmed cases and 4,849 deaths in China as of 18^th^ March 2021. Meanwhile, the number of confirmed COVID-19 cases and fatalities around the world has reached 121,719,887 and 2,687,905, respectively (2). The typical symptoms of COVID-19, including fever, cough, shortness of breath and pneumonia, are very common in mild cases, while acute respiratory distress syndrome (ARDS), acute cardiac injury and lymphocytopenia are also prevalent, especially in aged and critically ill patients, implying that SARS-CoV-2 affects various organs (3, 4).

It has been reported that SARS-CoV-2 interacts with human angiotensin-converting enzyme 2 (ACE2) *via* the spike protein for human to human transmission. Overwhelming data have shown that the ACE2 protein has a wide tissue distribution, including in the lung, liver, stomach, ileum, colon and kidney (5). Single-cell RNA sequencing of 13 human tissues showed that *Ace2* mRNA is mainly expressed in lung AT2 cells, liver cholangiocytes, colon colonocytes, esophagus keratinocytes, ileum epithelial cells (ECs), rectum ECs, stomach ECs and kidney proximal tubules (6). However, no data are available with regards to whether some types of immune cells or cells in the secondary lymphoid organs are positive for ACE2, or whether this leads to SARS-CoV-2 infection and subsequent lymphocytopenia in COVID-19 patients. Therefore, deciphering SARS-CoV-2 infection in immune organs is critical for understanding virus-mediated lymphocytopenia and possibly identifying methods to boost the number of lymphocytes and enhance host immunity.

In this report, we retrospectively reviewed the clinical data from 18 deceased COVID-19 patients who were admitted to the Huoshenshan Hospital in Wuhan, Hubei Province, from February 5^th^ to April 10^th^, 2020. To further decipher the mechanisms underlying the lymphocytopenia caused by SARS-CoV-2 infection, the secondary lymphoid organs, including the spleens and the hilar lymph nodes (LNs), from six additional COVID-19 patients with post-mortem examinations at the Jinyintan Hospital, another designated hospital in Wuhan, were also collected. We used hematoxylin & eosin (H&E) staining, transmission electronic microscopy (TEM) assay, RNA *in situ* hybridization (ISH), immunohistochemistry (IHC) and immunofluorescent double staining to visually assess the histopathology and viral presence in the spleen and the hilar LNs. Furthermore, we investigated the mechanisms of virus-mediated lymphocytopenia by directly infecting human primary macrophages and dendritic cells (DCs) with SARS-CoV-2 *in vitro*.

## Methods

### Data Collection and Definitions

Electronic medical records from 18 deceased COVID-19 patients who were admitted to the First Pneumonia Ward of Huoshenshan Hospital from February 5^th^ to April 10^th^, 2020 were collected and retrospectively analyzed. Patients died within 3 days of the date corresponding to data. Diagnosis of COVID-19 was based on the New Coronavirus Pneumonia Prevention and Control Program (6^th^ edition) published by the National Health Commission of China. All of the patients were laboratory-confirmed positive for SARS-CoV-2 by use of quantitative RT-PCR (qRT-PCR) of throat swab samples. Primers and probes targeting the SARS-CoV-2 nucleocapsid gene were used and the sequences were as follows: forward primer, 5′-TCAGAATGCCAATCTCCCCAAC-3′; reverse primer, 5′-AAAGGTCCACCCGATACATTGA- 3′; and the probe, 5′-CY5-CTAGTTAC ACTAGCCATCCTTACTGC-3′-BHQ1. Conditions for the amplifications were 50°C for 15 minutes and 95°C for 3 minutes, followed by 45 cycles of 95°C for 15 seconds and 60°C for 30 seconds. Three investigators (Dr. Yi Zhang; Dr. Qinghua Qiao and Dr. Bo Diao) directly collected the electronic medical records and independently reviewed the data collection forms to verify data accuracy.

### COVID-19 Post-Mortem Autopsies

The spleens and the hilar LNs from six COVID-19 patients with post-mortem examinations were collected, these deceased COVID-19 patients were admitted to Jinyintan Hospital in Wuhan, Hubei Province, China. Due to the special infection-control precautions for handling deceased subjects with COVID-19, post-mortem examination was performed in a designated pathology laboratory. The deceased patients were taken for post-mortem examination immediately (4 cases) or kept at -20°C until post-mortem analysis was carried out within 24 hours of death (2 cases). The spleens and the hilar LNs were collected by standard examination at autopsy. Collection of other medical data for these six cases with post-mortem examinations was executed by two investigators (C Tu and R Chen). The spleens and LNs were also collected from two trauma victim autopsies (male, 65 years old; female, 62 years old), one biopsy from an EBV-infected patient (male, 47 years old), and one biopsy from a patient with HBV-related acute-on-chronic liver failure (HBV-ACLF; male, 60 years old) who also manifested ARDS. This study was approved by the Ethics Commission of the Jinyintan Hospital (KY-2020-15.01).

### H&E Staining

Briefly, paraffin-embedded tissue blocks were cut into 2.5 μm slices and mounted onto poly-lysine-coated glass slides. Sections were incubated with haematoxylin for 5 min, and then after 1 min of dehydration with 100% alcohol, sections were further treated with eosin for 30 sec. Sections were then mounted and viewed using a light microscope (Zeiss Axioplan 2, Berlin, Germany). Histopathological results were independently assessed by two blinded investigators (Dr. Changsong Wang and Dr. Rong Chen).

### TUNEL Staining

Cellular apoptosis was measured using an *In Situ* Cell Death Detection Kit, POD, according to the manufacturer’s instructions (#11684817910, Roche, Berlin, Germany). The proportion of terminal deoxynucleotidyl transferase dUTP nick end labelling (TUNEL)-positive nuclei in spleens and LNs were determined through image analysis of the histological sections.

### IHC

The protocol for immunohistochemistry was published previously (7). Briefly, paraffin-embedded tissue blocks were cut into 2.5 μm sections and mounted on poly-l-lysine-charged glass slides. Sections were de-waxed and rehydrated, and antigen retrieval was performed by microwaving in 10 mM citrate buffer (pH 6.0). Endogenous peroxidase activity was blocked by incubation with a solution of 0.5% hydrogen peroxidase (H_2_O_2_) in 50% methanol for 10 min. The sections were then incubated in 2% BSA plus 0.1% Nonidet P-40 in PBS for 1h at RT to block nonspecific binding. Sections were then incubated overnight at 4°C with primary antibodies including anti-SARS-CoV-2 nucleocapsid protein (NP) (#clone ID: 019, 1:200, rabbit IgG1; Sino Biological, Beijing, China), anti-TRMPSS2 (#204314-T08, 1:100, rabbit IgG1; Sino Biological), anti-SARS-CoV-2 NP (#ab273434, 1:500, mouse monoclonal 6H3; Abcam, Shanghai, China), anti-SARS-CoV-2 spike glycoprotein (S) (#ab273433, 1:500, mouse monoclonal 1A9; Abcam), anti-ACE2 (#clone ID: 10108-RP01, 1:200, rabbit IgG1; Sino Biological), anti-FasL (#sc-834, 1:300, rabbit IgG1; Santa Cruz, San Francisco, CA, USA), anti-IL-1β (#ab9722, rabbit IgG1, Abcam), anti-IL-6 (#12153, Rabbit mAb, CST), rabbit IgG1 isotype (#10500C, 1:200; ThermoFisher, Waltham, MA, USA) or mouse IgG1 isotype (#02-6100, 1:200; ThermoFisher) antibodies. After washing, sections were incubated with the corresponding secondary antibodies (polyclonal goat anti-rabbit IgG/biotinylated, #00070204, 1:200, DAKO, Copenhagen, Denmark or polyclonal rabbit anti-mouse IgG/biotinylated, #00070978, 1:200, DAKO) for 1h at RT. The Vecta-stain ABC kit (#ZD0810, Vector Laboratories, San Diego, CA, USA) was used for the avidin-biotin complex method according the manufacturer’s instructions. Peroxidase activity was visualized with the DAB substrate kit (#ab64238; Abcam), and brown coloration of tissues represented positive staining. The sections were lightly counterstained with hematoxylin, dehydrated through an ethanol series to xylene, and mounted. Finally, sample sections were viewed using a light microscope (Zeiss Axioplan 2).

The proportion of TUNEL-, SARS-CoV-2 NP-, IL-6- or IL-1β- positive cells was determined by image analysis of histological sections. Photomicrographs from high power fields (hpf, original magnification, 23058 μm^2^) were captured and analyzed using Image-Pro Plus 5.0 software (Media Cybernetics, Silver Spring, MD). The numbers of SARS-CoV-2 NP-, IL-6 or IL-1β *per* hpf in the red pulp of spleens and in the marginal sinus of the lymph nodules of hilar LNs from each fatal COVID-19 cases were counted. Results were independently assessed by two blinded investigators (Dr. Changsong Wang and Dr. Rong Chen).

### Immunofluorescent Double Staining

Sections were incubated at 4°C overnight with primary mouse originated antibodies including anti-SARS-CoV-2 NP (#ab273434, 1:500, mouse IgG1; Abcam), anti-ACE2 (#MA5-31395, clone ID: CL4035, 1:400, mouse IgG1; Invitrogen), anti-CD68 (#MAB11303, clone KP1, 1:300, mouse IgG1; Bio-RAD), anti-CD169 (#346020, clone ID: 7-239, 1:200, mouse IgG1; Biolegend), anti-B220 (#MAB11301, clone ID: 123C3, 1:100, mouse IgG1; Bio-RAD), anti-CD11c (#ab254183, clone ID: KB90, 1:200, mouse IgG1; Abcam), and anti-Fas (#48095942, 1:200, mouse IgG1; ThermoFisher), and rabbit originated antibodies including anti-SARS-CoV-2 NP (#clone ID: 019, 1:200, rabbit IgG; Sino Biological), anti-IL-1β (#ab9722, rabbit IgG1, Abcam) and anti-IL-6 (#12153, Rabbit mAb, CST). After washing with PBS (3 washes, 5 min per wash), sections were incubated with Alexa Fluor^®^ 555-conjugated goat anti-rabbit IgG antibodies (#ab150078, 1:200, Abcam) or Alexa Fluor^®^ 488-conjugated goat anti-mouse IgG1 antibodies (#ab150078, 1:200, Abcam) for 1 h. Finally, the sections were incubated with 1 μg/ml DAPI (Sigma, St. Louis, MO, USA) for 10 min to stain the nuclei. Sections incubated with the appropriate isotype control antibodies and fluorescently labelled secondary antibodies were used as negative controls. Results were analyzed using fluorescence microscopy (Zeiss Axioplan 2).

### TEM Assay

Two hilar lymph nodes from 2 COVID-19 autopsies (case #1 and case #2) were collected and fixed with 2.5% glutaraldehyde in phosphoric buffer (pH 7.4), post-fixed with 1% osmate, dehydrated with gradient alcohol, embedded in Epon 812, and double-stained with uranium acetate and lead citromalic acid. The virus-like particles were observed under a JEM1200 transmission electron microscope (Jeol, Tokyo, Japan).

### Detection of SARS-CoV-2 Viral RNA by ISH

SARS-CoV-2 viral RNA *in situ* hybridization (ISH) in the spleens and LNs from three cases of fatal COVID-19 was performed using RNAScope^®^ Probe-V-nCoV2019-S (#848561, ACD, Newark, CA) directed against SARS-CoV-2 targeting 21631-23303 of NC_045512.2 and RNAScope^®^ Probe-V-nCoV2019-S-sense (#845701, ACD). A negative control probe targeting the bacterial gene diaminopimelate B (DapB, #310043, ACD) was utilized to analyse non-specific background, and a probe to the housekeeping gene peptidylprolyl isomerase B (PPIB) was used for RNA integrity (#313901, ACD) as positive control. The RNAScope^®^ 2.5 HD Reagent Kit-RED (#322350, ACD) was utilized per the manufacturer’s instructions. Briefly, the spleen and LN tissue retrieval was performed at 95°C for 20 min, followed by incubation with the RNAScope protease for 15 min at 40°C. Probes were added and hybridized was carried out for 3 h at 42°C using the following protocol: AMP1 3,3′-diaminobenzidine (DAB) was incubated for 30 min, AMP2 DAB for 15 min, AMP3 DAB for 30 min, AMP4 DAB for 15 min, AMP5 DAB for 30 min, and AMP6 DAB for 15 min, followed by incubation with DAB for 20 min. Sections were counterstained with periodic acid-Schiff. Here, SARS-CoV-2 viral RNA was also detected in the spleens of trauma victims. A similar protocol was also used to detect viral RNA in macrophages and DCs with or without SARS-CoV-2 infection *in vitro*.

### Cells and SARS-CoV-2 Infection *In Vitro*

Peripheral blood mononuclear cells (PBMCs) were isolated from fresh blood samples of healthy donors using human peripheral lymphocytes separation medium (#LTS1077-1, TBD, Tianjin Haoyang) according to recommended protocol. Isolated PBMCs were then allowed to adhere to cell plates pre-coated with poly-D-lysine. After 2 h of adhesion, non-adherent cells were washed away with pre-warm PBS. Adherent monocytes were cultured in RPMI-1640 (#C11875500BT, Gibco) supplemented with 10% FBS (#10099141C, Gibco) and 1% Penicillin-Streptomycin (#10378016, Gibco) at 37°C with 5% CO_2_ atmosphere. Monocytes-derived macrophages (MDMs) were differentiated by providing human granulocyte-macrophage colony-stimulating factor (hGM-CSF, #300-03, 50 ng/mL, Peprotech) for 6 days, while Monocytes-derived dendritic cells (MoDCs) were differentiated by adding hGM-CSF (#300-03, 50 ng/mL, Peprotech) and human interleukin-4 (hIL-4, #200-04, 25 ng/mL, Peprotech) for 6 days. The SARS-CoV-2 virus was provided by Prof. Liu reported previously (8). For virus infection, MoDCs and MDMs were replaced with fresh RPMI-1640 and then infected with SARS-CoV-2 at a MOI = 0.1 for 2 h. After virus absorption, cells were washed with pre-warmed RPMI-1640 twice and maintained in RPM1640 containing 2% FBS for 24 h before further measurement.

### Quantitative RT-PCR

Total RNA was extracted from SARS-CoV-2-infetced MDMs and MoDCs using TRIzol reagent (#15596026, Invitrogen) according to the manufacturer’s instructions. First-strand cDNA was synthesized with the PrimeScript RT reagent Kit with gDNA Eraser (#RR047A, Takara). The primers specific for target gene were designed by Primer 5.0 software and confirmed by using BLAST program in NCBI. The expression of target genes was quantified by using the TB Green Premix Ex Taq II (#RR820, Takara) on the CFX96 real-time PCR thermal cycle instrument (Bio-Rad). The expression of each gene was normalized to the expression of HPRT housekeeper gene, and the fold change of gene expression was further calculated using 2*^−ΔΔCt^* method. Primer sequences used were showed in **Table S3**.

### Statistical Analysis

For all analysis, two-tailed, unpaired Student’s t-tests with a 95% confidence interval performed on graphs generated in GraphPad Prism GraphPad Prism version 8.0 (GraphPad Software, Inc., San Diego, CA, USA) were used. Error bars represent the SEM. *p*< 0.05 was considered a statistically significant difference. All results shown are representative of at least three separate experiments.

## Results

### Lymphocytopenia and Inflammation Are Common in Deceased COVID-19 Patients

We retrospectively analyzed the cases of 18 deceased COVID-19 patients who were admitted to the Intensive Care Unit (ICU) of Huoshenshan Hospital in Wuhan, Hubei Province. These patients included 14 (77.8%) males and 4 (22.2%) females, with an average age of 74.83 years. A total of 13 (72.2%) patients were over 70 years old and 11 patients had at least one underlying disease, including hypertension (n = 9; 50.0%), type-I diabetes (n = 4; 22.2%) (**Table S1**). All of these patients suffered from ARDS based on available arterial blood gas (ABG) analysis carried out when their data was collected. Other clinical symptoms included dyspnea (n = 16; 88.9%), fever (n = 14; 77.8%), cough (n = 11; 61.1%), fatigue (n = 10; 55.6%), respiratory failure (n = 7; 38.9%), myalgia (n = 3; 16.7%) and expectoration (n = 2; 11.1%). Less common complications included respiratory failure (n = 7; 38.9%), acute cardiac injury (n = 6; 33.3%), acute kidney injury (n = 5; 27.8%), secondary infection (n = 4; 22.2%) and acute liver injury (n = 1; 5.6%). These patients were given empirical antimicrobial treatment including moxifloxacin and/or cephalosporin, as well as antiviral therapy like oseltamivir and/or ganciclovir. All cases received corticosteroid (methylprednisolone) during the course of hospitalization. A total of 16 (88.9%) cases required non-invasive mechanical ventilation continuous positive airway pressure (CPAP). The average number of hospital days was 41.56 days from the onset of illness to death (**Table S1**).

We next retrospectively reviewed the clinical and immunological data of these patients, and all patients died within 3 days of the date corresponding to data. The mean absolute numbers (13.51 x 10^9^/L) of white blood cells (WBC) in these patients were 1.42 folds higher than the upper limit of normal range (ULN). A total of 17 patients had lower blood platelet counts (mean value: 99.76 x 10^9^/L), erythrocyte counts (mean value: 3.04 x 10^12^/L) and serum hemoglobin concentration (mean value: 95.78 g/L) than the corresponding lower limit of normal ranges (LLN). However, 14 (77.8%) patients had both high percentage (mean value: 88.31%) and increased absolute number (mean value: 12.33 x 10^9^/L) of neutrophils (**Table 1**). A total of 17 (94.4%) patients had lymphocytopenia and the mean lymphocyte counts were 0.64 x 10^9^/L, which was below the LLN. Importantly, depletion of T cells (mean value: 3.95 x 10^8^/L) was more prominent than that of B cells (mean value: 11.31 x 10^6^/L) in these patients (**Table 1**), demonstrating that lymphocytopenia is the main feature of severe COVID-19 disease.

**Table 1:** Laboratory findings of perished patients with COVID-19.

	Cells in blood												Cytokines							Alarmins						
Cases	WBC, ×10^9^	Platelet counts, x10^9^	Erythrocytecounts, x10^12^	Hemoglobin(g/L)	Neutrophils (%)	Neutrophil counts, x10^9^	Monocytes (%)	Monocyte counts, x10^9^	Lymphocytes (%)	Lymphocyte count, x10^9^/L	T cell counts, x 10^8^/L	B cell counts, x 10^6^/L	IL-6 (pg/mL)	IL-1β (pg/mL)	IFN-γ (pg/mL)	IL-17 (pg/mL)	IL-8 (pg/mL)	IL-10 (pg/mL)	TNF-α(pg/mL)	CRP (mg/mL)	D-Dimer (mg/L)	DBIL (umol/L)	Urea (umol/L)	LDH (IU/L)	HBDH (IU/L)	PCT (ng/ml)
#1	19.8	14	3	94	95.4	18.89	1.1	0.21	2.4	0.48	4.10	5.18	7333.97	13.67	80.83	13.95	#####	114.46	3.91	214.3	5.43	376	16.17	649.7	593.5	12.25
#2	9.3	211	2.64	94	89.9	8.31	4.4	0.41	5.6	0.52	3.74	10.09	298.84	4.05	1.28	3.04	49.89	11.26	0.03	15.75	1.01	8.2	13.83	389.7	304.3	0.11
#3	10.6	110	2.9	90	93.8	9.94	2.1	0.22	4.1	0.44	2.05	5.76	3720.89	13.67	2.02	4.11	559.41	24.72	1.30	145.5	4.01	9.1	18.68	446.6	391.6	11.53
#4	11.9	123	2.95	86	79.9	9.49	8.3	0.99	8	0.94	6.80	4.23	83.22	3.80	3.89	4.09	25.25	4.51	3.13	61.19	1.24	4	13.85	294	187.6	0.14
#5	3.1	25	3.11	102	72.9	2.26	2.3	0.07	24.4	0.75	2.77	37.28	2475.63	0.17	4.93	3.45	369.23	12.83	2.64	57.92	2.05	22.8	23.29	312.4	272.9	1.15
#6	13.9	229	3.55	109	87.4	12.14	4.6	0.65	6.5	0.9	6.47	5.76	124.25	1.18	6.94	5.81	121.20	6.64	2.00	80.26	3.09	2.3	37.68	311.2	251.4	0.7
#7	5.5	81	3	94	87.5	4.81	5.3	0.29	5	0.28	1.63	1.40	1466.34	2.42	4.57	3.24	18.37	2.74	3.13	101.8	2.52	21.8	18.7	341	293.1	2.15
#8	11.7	120	3.27	102	91.9	10.75	2.7	0.32	5	0.58	3.62	13.40	14761.35	1.18	4.57	3.31	25.25	4.10	2.00	81.3	3.73	39.3	11.73	220.5	166.9	1.67
#9	35.8	137	2.97	91	96	34.34	0	0	2.5	0.91	6.27	10.65	581.89	17.29	10.82	4.93	369.23	43.80	5.24	167.23	1.11	43.7	24.06	330.4	260.3	3.75
#10	76.2	183	2.85	90	96.5	73.51	1.8	1.36	1.0	0.73	4.42	3.50	65.28	0.00	26.98	4.54	96.81	6.64	2.41	113.35	4.63	11.1	27.90	236.50	155.40	3.22
#11	12.5	43	2.35	78	71.0	8.84	14.1	1.76	13.8	1.72	10.03	17.72	29.93	17.29	11.93	6.05	837.69	35.79	7.59	25.80	2.43	2.9	10.52	110.90	94.50	0.29
#12	0.2	14	3.92	106	29.5	0.06	1.1	0.00	67.6	0.14	0.23	10.93	529.50	0.00	2.96	3.60	92.80	7.42	2.00	101.64	1.71	96.6	5.56	352.30	311.20	5.35
#13	33.6	188	3.81	118	95.1	31.88	0.9	0.31	2.7	0.91	6.03	23.30	5332.71	35.47	45.05	3.44	151.32	20.98	4.21	177.01	1.25	17.9	4.99	534.80	408.60	2.98
#14	36.6	235	2.43	74	97.4	35.61	1.1	0.39	1.4	0.52	3.78	1.77	395.59	15.43	35.50	3.84	251.92	26.11	4.57	64.70	1.27	7.8	2.58	370.90	289.70	0.36
#15	11.5	10	2.25	72	95.1	10.96	2.1	0.24	2.5	0.29	2.30	3.86	356.30	0.00	29.67	2.85	61.80	6.09	2.41	48.79	2.32	34.2	3.80	>1000.00	>1000.00	1.98
#16	6.8	40	2.64	82	89.5	6.05	1.7	0.12	8.8	0.60	2.03	18.12	37.23	29.24	9.27	4.93	717.18	23.95	5.97	199.93	5.37	10.2	12.85	523.60	441.90	0.39
#17	8.6	57	4.34	115	96.3	8.28	1.5	0.13	2.1	0.18	1.02	4.95	371.2	7.86	2.93	4.06	52.75	52.56	1.23	122.41	12	5.8	9.33	347.90	294.20	0.56
#18	21.3	64	2.24	68	90.6	19.31	4.3	0.92	3.4	0.72	3.79	25.92	415.90	5.94	0.56	2.34	92.04	7.26	0.35	161.53	2.85	2.3	23.61	351.30	297.50	1.65
Mean	13.51	99.76	3.04	95.78	88.30	12.33	3.42	0.35	7.06	0.64	3.95	11.32	2869.40	9.37	15.82	4.53	283.99	22.88	3.01	102.81	3.31	58.58	19.78	#####	#####	3.40
Normal range	3.5-9.5	125-350	4.0-5.8	120-170	40-75 %	1.8-6.3×10^9^	3-10 %	0.1-0.6×10^9^	20-50 %	1.1-3.2×10^9^	9.5-28x10^8^	5-18x10^6^	< 5.4	<12.4	<23.1	<21.4	<20.6	<12.9	<16.5	0-4	<0.55	0-8	3.1-8	120-250	72-182	0-0.05

HBDH, α-hydroxybutyrate dehydrogenase; CRP, c-reactive protein; DBIL, direct bilirubin; LDH, lactate dehydrogenase; PCT, procalcitonin.

The dysregulation of pro-inflammatory cytokines can induce lymphocyte depletion (3, 9), and high levels of serum IL-6 and IL-8, as well as IL-10, were observed in these fatal cases. In particular, the average serum IL-6 concentration reached 2869.4 pg/mL, which is 531.37 folds higher than the ULN. The mean concentration of serum IL-8 was 283.99 pg/mL, which is 22.01 folds above the ULN. Additionally, the mean serum level of IL-10 was also 1.77 folds higher than the ULN. Although some COVID-19 cases have very high serum concentration of IL-17, IL-1β and TNF-α, the average levels of these cytokines did not change dramatically compared to normal values (**Table 1**). Furthermore, enhancement of C-reactive protein (CRP, mean value 102.81 mg/L, 25.7 folds above the ULN), D-dimer (mean value 3.31 mg/L, 6.02 folds above the ULN), direct bilirubin (DBIL, 58.58 μmol/L, 7.32 folds above the ULN), blood urea nitrogen (urea, 19.78 μmol/L, 2.47 folds above the ULN), lactate dehydrogenase (LDH, 366.17 IU/L, 1.46 folds above the ULN), α-hydroxybutyrate dehydrogenase (HBHD, 302.4 IU/L, 1.66 folds above the ULN) and procalcitonin (PCT, 3.4 ng/mL, 68 folds above the ULN) was also observed in most cases (**Table 1**). However, serum concentrations of other alarmins were not significantly different from normal values (**Table S2**). Collectively, these data suggest that lymphocytopenia and systemic inflammation are very common in patients that succumb to COVID-19.

### SARS-CoV-2 Destroys Human Spleens and LNs

**Figure 1:**
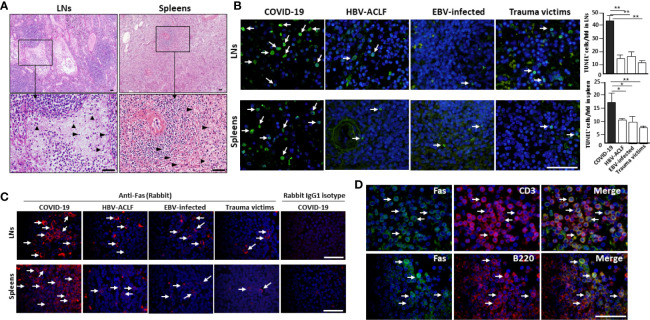
SARS-CoV-2 destroys human spleen and LN tissues. The spleens and hilar LNs were collected from one representative fatal COVID-19 (case #2), HBV-ACLF, EBV-infected patients and trauma victim, **(A)** histopathology was compared by H&E staining, arrow heads show apoptotic cells; **(B)** cell apoptosis was detected by *in situ* TUNEL staining (left), with statistical analysis of apoptotic cells (right). Arrows indicate positive cells, *p < 0.05 and **p < 0.01; **(C)** immunofluorescent staining analysis of Fas expression; **(D)** immunofluorescent double staining analysis of Fas expression by CD3^+^ T cells and B220^+^ B cells in the hilar LNs. Scale bar = 50 μM arrows indicate double positive cells.

In addition to inducing circulating lymphocyte depletion by enhancing inflammation, SARS-CoV-2 might also promote lymphocytopenia by directly destroying human secondary lymphoid organs, since this virus is well known to be highly cytotoxic. Therefore, the hilar LNs and spleens were collected from warm autopsies of six COVID-19 subjects. All of these deceased patients manifested severe lymphocytopenia, as demonstrated by extremely low lymphocyte counts. They were also given empirical antimicrobial treatment and antiviral therapy, and two cases even received corticosteroids during their course of hospitalization, none of them received extracorporeal membrane oxygenation (ECMO) treatment. Other clinical characteristics of these 6 deceased patients are shown in **Table 2**. H&E staining of lung tissues confirmed that the alveolar wall was thickened, and that telangiectasia, ecchymosis and strong inflammatory cell infiltration occurred, suggesting that SARS-CoV-2 worsens lung damage (**Figure S1**). Surprisingly, acute tissue damage and lymphocyte reduction occurred in all of the assessed hilar LNs and spleens. The LNs were congested, hemorrhagic and accompanied by subcapsular sinus histiocytosis, some cases also showed an enlargement of the abdominal lymph nodes, which had reduced number of germinal centers. Moreover, the lymph follicles and paracortical areas in some patients were not fully identifiable, with necrotic and apoptotic lymphocytes being widely distributed. Similarly, all of the spleen tissues exhibited atrophy of the white pulp, with a complete loss in some cases. The red pulp was markedly congested, hemorrhagic, and the total lymphocyte counts were also significantly reduced (**Figure 1A**, **Table 2**), whereas, the spleens and LNs from trauma victims and HBV-ACLF patients manifested normal morphologic structures. By contrast, the spleens and LNs from EBV-infected patients showed smaller lymphoid follicles, T zone proliferation and an enhancement of immunoblastic cells (**Figure S2**), demonstrating that SARS-CoV-2 causes severe damage in human spleens and LNs that is different from that in EBV and HBV infection.

**Table 2:** The pathologic findings from COVID-19 patients undergoing postmortem examination.

Case	#1	#2	#3	#4	#5	#6
**Coexisting disorder**						
Hypertension	>10 yr	>10 yr	–	>10 yr	>5 yr	–
Coronary disease	–	>10 yr	–	–	–	–
Gout	–	–	–	–	Yes	–
**Cause of death**	ARDS	ARDS	ARDS	ARDS	ARDS	ARDS
**SARS-CoV-2 confirmed by PCR**	Yes	Yes	Yes	Yes	Yes	Yes
**Corticosteroid**	Yes	**-**	**-**	**-**	**-**	Yes
**H&E staining**						
**Spleens**						
Congestion and hemorrhage	++	+++	+++	++	+++	++
WP destruction or atrophy	++	+++	+++	++	+++	++
Marginal areas disappearance	Yes	Yes	Yes	Yes	Yes	Yes
Lymphocyte depletion	Yes	Yes	Yes	Yes	Yes	Yes
Splenic nodule atrophy	Yes	–	–	–	–	–
Interstitial fibrosis	Yes	–	–	–	–	–
Reduced number of GC	–	Yes	–	–	Yes	Yes
**LNs**						
Congestion and hemorrhage	++	+++	+++	++	+++	++
lymph follicle disappearance	Yes	Yes	Yes	Yes	Yes	Yes
histiocyte hyperplasia	Yes	Yes	Yes	Yes	Yes	Yes
Eltangiectasia	Yes	Yes	Yes	Yes	Yes	Yes
Lymphocyte depletion	Yes	Yes	Yes	Yes	Yes	Yes
**Immunohistochemistry (Nubers of positive cells/hpf)**						
SARS-CoV-2 NP in spleens	72.5±10.43	15.75±2.75	71.83±17.58	16.75±4.35	63.5±27.74	67.25±14.37
SARS-CoV-2 NP in LNs	214.3±33.1	119.5±31.2	59.25±17.21	60.5±21.16	80.25±21.71	34±12.4
IL-1β in spleens	257±18.06	136.5±22.53	99.5±16.24	151.2±26.91	187±30.93	67.25±13.68
IL-1β in LNs	135.5±38.86	63.5±12.9	56±5.30	41±8.44	96.25±17.72	45±11.08
IL-6 in spleens	142.8±16.07	125.2±24.26	139.4±26.86	118.6±9.30	66.5±4.66	113.5±41.02
IL-6 in LNs	10.25±2.56	19.5±4.01	57.25±14.99	21±2.48	9.75±1.38	77.75±10.91
**SARS-CoV-2 viral RNA**						
Probe-V-nCoV2019-S in spleens	undetected	72.18±10.03	undetected	undetected	undetected	61.20±11.23
Probe-V-nCoV2019-S in LNs	undetected	17.5±6.05	undetected	undetected	undetected	10.2±3.48
Probe-V-nCoV2019-S-sense in spleens	undetected	85.44±12.11	undetected	undetected	undetected	65.29±8.36
Probe-V-nCoV2019-S-sense in LNs	undetected	10.5±5.31	undetected	undetected	undetected	9.35±4.27
**Viral particles by TEM**	Yes	Yes	undetected	undected	undetected	undetected

yr, years; WP, White plup; GC, Germinal center; LN, Lymph node; TEM, Trasmision Electron Microscope.

# Expression levels: Negative:-; Moderate: ++; Severe: +++; Extremely severe ++++.

Vrial NP antigen was detected by anti-SARS NP antibodies (clone ID: 019, rabbit IgG; Sino Biological).

Immunohistochemistric data are showed by Mean±S.E.M

*In situ* TUNEL staining showed that spleens and hilar LNs from COVID-19 autopsies manifested strong lymphocyte apoptosis, whereas, apoptotic cells in tissues from age- and gender-matched HBV-ACLF and EBV-infected patients and trauma victims were nearly undetectable (**Figure 1B**). Cell apoptosis is primarily mediated by Fas and Fas ligand (FasL) signaling (10). IHC showed that spleen and LN tissues from COVID-19 patients and controls have similar levels of FasL expression (**Figure S3**). Conversely, sections from COVID-19 autopsies manifested significantly higher levels of Fas than those in controls (**Figure 1C**). Immunofluorescent double staining illustrated that Fas was expressed on the surface of CD3^+^ T cells and B220^+^ B cells in the hilar LNs from COVID-19 autopsies (**Figure 1D**), suggesting that SARS-CoV-2 promotes Fas-mediated T and B cell apoptosis in spleens and LNs.

### Identification of SARS-CoV-2 RNA and Coronavirus-Like Particles in Spleens and LNs

**Figure 2:**
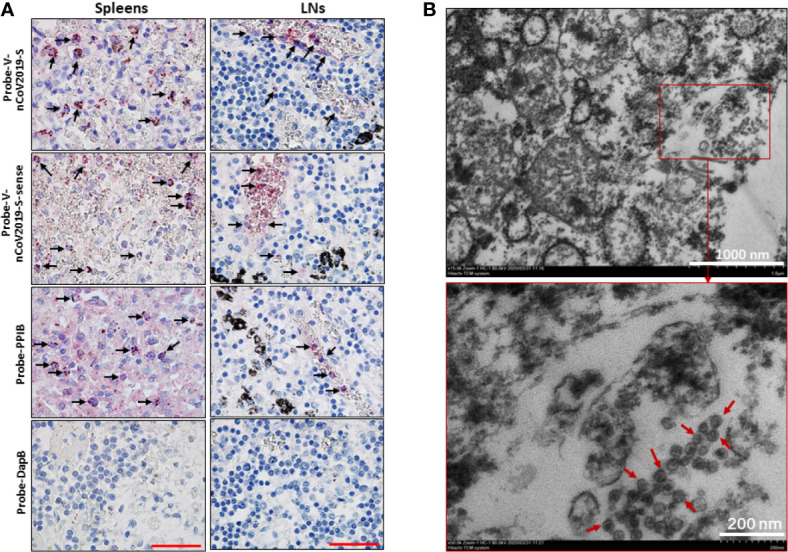
SARS-CoV-2 was identified in spleens and LNs. The spleen and hilar LNs from one representative COVID-19 post-mortem case (case #2) were collected, **(A)** SARS-CoV-2 viral RNA was detected by RNA ISH, the positive reactivity for the housekeeping gene PPIB was used as positive control. Scale bar = 50 μM, arrow indicates positive cells. **(B)** TEM assay showed that some coronavirus-like particles with diameters of 50~100 nm were observed in hilar LNs. Arrows indicate coronavirus-like particles.

To confirm that SARS-CoV-2 directly infects human spleens and LNs, leading to lymphocyte depletion *in vivo*, RNA ISH was performed using RNAScope probes directed against the SARS-CoV-2 *S* gene, targeting 21631-23303 of NC_045512.2 base pairs (RNAScope^®^ Probe-V-nCoV2019-*S*, #848561, ACD, Newark, CA), and RNAScope^®^ Probe-V-nCoV2019-*S*-sense (#845701, ACD). Results showed that spleen and hilar LN tissues from COVID-19 autopsies manifested positive signals for Probe-V-nCoV2019-*S*. In spleens, the positive cells were distributed throughout the whole tissue, especially in red pulp, whereas in the hilar LNs, the positive reaction was found primarily in cells within marginal sinus of the lymph nodules, demonstrating that SARS-CoV-2 directly infects some cells within human spleens and LNs. Interestingly, cells in the spleens and hilar LNs were also positive for Probe-V-nCoV2019-*S*-sense, illustrating that SARS-CoV-2 has the capacity to replicate *in vivo*. Here a negative control probe to the bacterial gene diaminopimelate B (DapB) and a positive control probe for RNA integrity to the housekeeping gene peptidylprolyl isomerase B (PPIB) were also included (**Figure 2A**, **Table 2**). However, the spleens from trauma victims were negative for Probe-V-nCoV2019-*S* (**Figure S4**). Collectively, these data demonstrate that SARS-CoV-2 directly infects some cells in human spleens and LNs.

We next investigated whether human secondary lymphoid organs have SARS-CoV-2 virus by transmission electron microscope (TEM) assay. Two hilar LNs of 1.0~1.5 cm in diameter from two autopsies of COVID-19 subjects (Case #2 and #6) were used to detect virions. The results showed that cells in both hilar LNs were markedly swollen with an expansion of mitochondria and lysosomes. The rough endoplasmic reticulum (RER) and smooth endoplasmic reticulum (SER) were also dilated significantly. Interestingly, **c**oronavirus-like particles were identified in the cytoplasm. The diameter of the virus-like particles varied from approximately 50~100 nm, the interior of virus particles showed electron-dense granular interiors, which are believed to be nuclear capsid. Moreover, virus-like particles show relative uniformity in size, making them different from endosomal structures or other cell trafficking micro-vesicles (**Figure 2B**). These combined data confirm that SARS-CoV-2 directly infects human spleens and hilar LNs.

### ACE2 Is Expressed by Macrophages and DCs in Spleens and LNs

**Figure 3:**
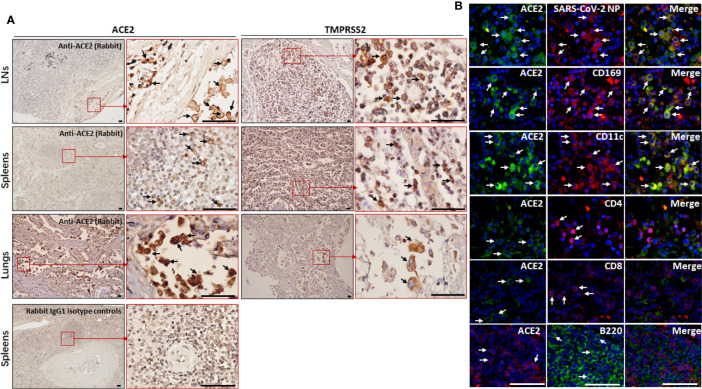
The expression of ACE2 and TMPRSS2 was observed by macrophages and DCs in spleens and hilar LNs. The spleen, hilar LNs and lung tissues from one representative COVID-19 post-mortem case (case #2) were collected, **(A)** the expression of ACE2 and TMPRSS2 was detected by IHC, arrows indicate positive cells; **(B)** immunofluorescent double staining analysis of ACE2 antigen on indicated cells within hilar LNs, scale bar= 50 μM, arrows indicated double positive cells and arrow heads showed single positive cells.

SARS-CoV-2 uses the SARS-CoV receptor ACE2 for entry and the serine protease TMPRSS2 for S protein priming (11). We then investigated the expression of ACE2 and TMPRSS2 in the spleens and hilar LNs. IHC showed that both ACE2 and TMPRSS2 antigens could be observed in the spleens and LNs from COVID-19 autopsies. In the LNs, the expression of ACE2 was mainly on cells within the medulla, especially cells in marginal sinus of the lymph nodules, whereas in the spleens, ACE2^+^ cells were found primarily in the splenic red pulp. TMPRSS2 has similar expressional characteristics to ACE2 in these tissues. Here the expressions of ACE2 and TMPRSS2 in lung tissues from COVID-19 patients were used as positive controls, and sections incubated with rabbit IgG1 antibodies were used as isotype controls (**Figure 3A**).

The hilar LNs from COVID-19 autopsies were selected to detect which cell subsets are positive for ACE2, and immunofluorescent double staining showed that ACE2 is expressed by SARS-CoV-2 NP positive cells, CD68^+^ macrophages and CD11c^+^ DCs, while it is absent on CD4^+^ and CD8^+^ T cells and B220^+^ B cells (**Figure 3B**). Lymphoid tissue resident CD169^+^ macrophages function as gatekeepers that have the capacity to capture blood- and lymph-borne pathogens, and these CD169^+^ macrophages can also support viral replication (12). Immunofluorescent double staining further confirmed that the ACE2 antigen could be observed on CD169^+^ macrophages in LNs from COVID-19 autopsies (**Figure 3B**). Collectively, these results demonstrated that ACE2 is expressed by macrophages and DCs in spleens and LNs.

### SARS-CoV-2 Directly Infects Human Macrophages and DCs

**Figure 4:**
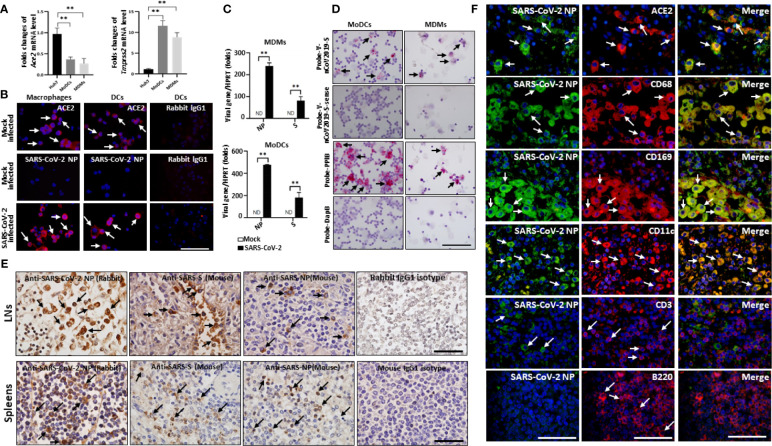
SARS-CoV-2 directly infects human macrophages and DCs. Human MDMs and MoDCs were mock infected or infected with SARS-CoV-2 (MOI =1.0) for 2h, after virus absorption, cells were washed and continuously cultured for additional 24h, **(A)**The expression of endogenous ACE2, TMPRSS2 were evaluated in MDMs, MoDCs and Huh7 cells, **p < 0.01. **(B)** The expression of ACE2 and SARS-CoV-2 NP antigen was detected by immunofluorescent staining; **(C)** qPCR analysis of SARS-CoV-2 viral RNA, **p < 0.01. **(D)** the presence of SARS-CoV-2 viral RNA was detected by RNA ISH, the reactivity for PPIB was used as positive control; The spleens and hilar LNs from one representative COVID-19 post-mortem (case #3) were collected, **(E)** sections were incubated with primary anti-SARS-NP antibodies (clone ID: 019, rabbit IgG; and ab273434, mouse monoclonal 6H3), anti-SARS-S antibodies (ab273433, mouse monoclonal 1A9), or with mouse or rabbit IgG1 control antibodies, and the expression of viral NP and S antigens were analyzed by IHC, scale bar = 50 μM, arrows indicate positive cells; **(F)** The hilar LNs were incubated with primary anti-SARS-NP antibodies (clone ID: 019, rabbit IgG) and other mouse derived antibodies, SARS-CoV-2 NP antigen in indicated cells was analyzed by immunofluorescent double staining. Scale bar = 50 μM, arrows indicated double positive cells and arrow heads showed single positive cells.

Our data above demonstrated that only macrophages and DCs within spleens and hilar LNs are positive for ACE2, suggesting SARS-CoV-2 might infect macrophages and DCs in these tissues. To confirm this possibility, peripheral blood mononuclear cells (PBMCs) from normal healthy patients were induced to develop into monocytes-derived macrophages (MDMs) and monocytes-derived DCs (MoDCs) *in vitro*. The expression of *Ace2 and Tmprss2* genes was observed in both kinds of cells (**Figure 4A**). Immunofluorescent staining also showed that both MDMs and MoDCs are positive for ACE2 protein (**Figure 4B**). These cells were further infected with SARS-CoV-2 at a multiplicity of infection (MOI) of 0.1 for 2h, after virus absorption, cells were washed and continuously cultured for additional 24h. Importantly, SARS-CoV-2-infected cells manifested high levels of SARS-CoV-2 NP antigen, whereas mock controls were absent of NP expression (**Figure 4B**). qPCR confirmed that SARS-CoV-2 viral RNA was present in infected but not mock control cells (**Figure 4C**). RNA ISH showed that cells with SARS-CoV-2 rather mock controls had strong viral RNA, however, the fact that Probe-V-nCoV2019-*S*-sense was negative in infected cells indicated SARS-CoV-2 might not be able to replicate in MDMs and MoDCs *in vitro* (**Figure 4D**). Our current result was similar to Yuen’s observations (13), suggesting that MDMs and MoDCs are susceptible to SARS-CoV-2 infection but seem to disrupt the effective replication of infectious virus.

We next examined whether SARS-CoV-2 infects macrophages and DCs in spleens and LNs *in vivo*, and the expression of SARS-CoV-2 specific NP and S antigens was examined. Two clones of anti-SARS-CoV-2 NP antibodies (#clone ID: 019, Rabbit polyclonal antibodies, Sino Biological, Beijing, China; #ab273434, mouse monoclonal 6H3, Abcam, Cambridge, UK) and one clone of an anti-SARS-CoV-2 S antibody (#ab273433, mouse monoclonal 1A9, Abcam) are commercially available at this time. IHC showed that both SARS-CoV-2 NP and S antigens were present in the spleens and hilar LNs of all six COVID-19 autopsies. In the spleen, viral NP^+^ cells were primarily distributed in the red pulp, whereas positive cells were occasionally observed in white pulp. In the LNs, the SARS-CoV-2 NP^+^ cells were primarily seen in cells within the marginal sinus of the lymph nodules, and NP^+^ cells were seldom found in germinal centers (**Figure 4D**). By contrast, SARS-CoV-2 NP and S antigens were absent in sections from trauma victims, and HBV-ACLF and EBV-infected patients (**Figure S5**). SARS-CoV-2 NP^+^ cells in the red pulp of spleens and in the marginal sinus of the lymph nodules of hilar LNs from each fatal COVID-19 cases were calculated by Image-Pro Plus software, and the results were showed in **Table 2**.

We then performed additional staining to identify the cell types that were positive for SARS-CoV-2. Immunofluorescent double staining of LNs from COVID-19 autopsies confirmed that the majority of SARS-CoV-2 NP antigen is found in ACE2^+^ cells, including CD68^+^ and CD169^+^ macrophages and CD11c^+^ DCs, whereas CD3^+^ T cells and B220^+^ B cells were negative for NP protein (**Figure 4E**), demonstrating that SARS-CoV-2 directly infects DCs and macrophages in spleens and LNs.

### SARS-CoV-2 Triggers IL-6 and IL-1β Production by Macrophages and DCs

**Figure 5:**
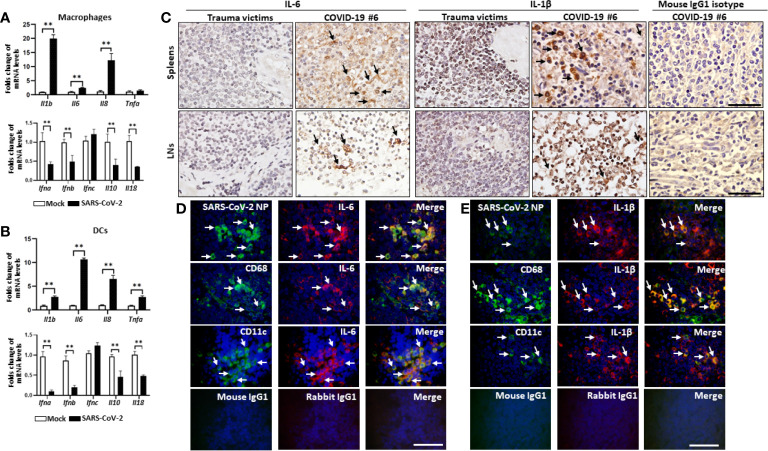
SARS-CoV-2 triggers IL-1β and IL-6 production by macrophages and DCs. Human MDMs and MoDCs were mock infected or infected with SARS-CoV-2 (MOI= 1.0) for 2h, after virus absorption, cells were washed and continuously cultured for 24h, the transcription of the indicated genes in **(A)** MDMs and **(B)** MoDC was detected by qRT-PCR, **p < 0.01. The spleens and LNs from one representative COVID-19 patient post-mortem (case #5) and trauma victim were collected, **(C)** the expression of IL-6 and IL-1β was analyzed by IHC, scale bar= 50 μM, arrows indicate positive cells; Sections from the hilar LNs were selected to analyze the secretion of **(D)** IL-6 and **(E)** IL-1β from the indicated cells by immunofluorescent double staining. Scale bar= 50 μM, arrows indicate double positive cells.

Our data above showed that these deceased COVID-19 patients had higher serum levels of some pro-inflammatory cytokines (**Table 1**), however, the original source of these cytokines is unclear. We speculated whether SARS-CoV-2 can directly trigger cytokine secretion from infected cells. MDMs and MoDCs were infected with SARS-CoV-2 (MOI=0.1, 2h) *in vitro* and cells were collected after 24h to detect the transcription of pro-inflammatory genes. Interestingly, qRT-PCR showed that both macrophages and DCs had significantly higher levels of *Il6, Il8* and *Il1b* gene transcription than mock infected controls. SARS-CoV-2 also promoted the transcription of *Tnfa* in MoDCs, but not MDMs. By contrast, the transcription of type-I interferons (IFN-I), like *Ifna* and *Ifnb*, was dramatically deceased in infected cells compared to mock cells (**Figures 5A, B**), suggesting that SARS-CoV-2 can promote the transcription of *Il6*, *Il8* and *Il1b* in both DCs and macrophages.

Pro-inflammatory cytokines IL-6 and IL-1β can induce cell apoptosis and pyroptosis, thus they may accelerate lymphocyte depletion *in vivo*. We next examined whether SARS-CoV-2 infection induces the expression of IL-6 and IL-1β in spleens and hilar LNs. IHC showed that high levels of IL-6 and IL-1β could be observed in the spleens and the LNs from COVID-19 autopsies, whereas, sections from trauma victims were absent of IL-6 and IL-1β expression (**Figure 5C**, **Table 2**). Immunofluorescent double staining showed that IL-6 and IL-1β are primarily produced by SARS-CoV-2 NP^+^ cells, including CD68^+^/CD169^+^ macrophages and CD11c^+^ DCs (**Figures 5D, E**). Collectively, these results suggest that SARS-CoV-2 can trigger macrophages and DCs to secrete IL-6 and IL-1β, which might directly decimate lymphocytes following infection *in vivo*.

## Discussion

Lymphocytopenia is one of the most prominent clinical presentations of immune damage caused by SARS-CoV, Middle East Respiratory Syndrome (MERS)-CoV and SARS-CoV-2 infection. SARS-CoV has been shown to significantly decrease CD4^+^ and CD8^+^ T cell counts by inducing inflammation, whereas, MERS-CoV has been illustrated to efficiently infect T cells and induce T cell apoptosis leading to lymphocytopenia (14–16). We here retrospectively reviewed the clinical and immunological data of 18 fatal COVID-19 cases, and the results showed that these patients had severe lymphocytopenia together with high serum concentrations of inflammatory cytokines IL-6, IL-8 and IL-10. Many routine laboratory tests, such as ferritin, lactate dehydrogenase and D-dimer, were elevated in severe and extremely severe COVID-19 patients (17). Similar to this work, high D-dimer levels were observed in all perished COVID-19 patients, reflecting a hypercoagulable state within these patients. Fourteen cases of these fatal patients also manifested higher urea levels compared to normal values, suggesting that SARS-CoV-2 affects renal function. Moreover, over 90% of these perished patients had increased α-hydroxybutyrate dehydrogenase, lactate dehydrogenase and creatine kinase activity, demonstrating that SARS-CoV-2 triggers myocardial injury. Alanine aminotransferase activity and aspartate aminotransferase activity were used to evaluate liver function, and we found 5 cases of these patients have liver function injury due to very high concentration of these mediators. Collectively, these data demonstrated that the cause of COVID-19 patients’ death is due to SARS-CoV-2-mediated multiple organ damage, cytokine storm and lymphocytopenia.

SARS-CoV-2 mainly transmits into the lung through respiratory tracts, it also distributes to heart, vessels, liver and kidney *etc.* A pathological report of three COVID-19 cases by minimal invasive autopsies illustrated that decreased numbers of lymphocytes, cell degeneration and necrosis were observed in the infected spleens (18)**. **Another study showed that SARS-CoV-2 infection induces hemophagocytic lymphohistiocytosis (19), suggesting that spleen and LNs may be directly attacked by SARS-CoV-2. The present results from 6 cases of COVID-19 autopsies showed that SARS-CoV-2 viral RNA and coronavirus-like particles could be seen in the spleens and hilar LNs. Interestingly, *in situ* immunohistochemistry illustrated that SARS-CoV-2 NP and S antigens were observed in cells within spleens and hilar LNs. Immunofluorescent double staining further demonstrated that SARS-CoV-2 NP antigen was restricted to ACE2-positive macrophages and DCs within spleens and LNs *in vivo*, suggesting that SARS-CoV-2 may employ ACE2 receptor to facility viral entry into macrophages and DCs. To the best of our knowledge, this is the first report of SARS-CoV-2 directly infecting human secondary lymphoid organs. Recent work of Wang *et al.* revealed that SARS-CoV-2 can infect T cells, although its ability to replicate in T cells is still inconclusive (20). However, we did not find that T or B cells were positive for ACE2, and SARS-CoV-2 NP antigen was absent from T and B cells, demonstrating that this virus does not have the capacity to directly infect and decimate lymphocytes in secondary lymphoid organs.

Innate immune cells in spleens undergo reorganization into “hierarchical clusters” that allow for the initiation and progression of an effective immune response against infections. Macrophages and DCs are sentinel cells for innate and adaptive immunity that affect the pathogenesis of SARS-CoV and MERS-CoV infection (21, 22). Furthermore, macrophages expressing high levels of CD169 (Siglec-1 or sialoadhesin), constitute a minor macrophage population present in lymphoid tissues. CD169^+^ macrophages are situated on top of B cell follicles, bordering the marginal sinus in the spleen and the subcapsular sinus (SCS) in the LNs, where they are also known as metallophilic marginal zone macrophages and SCS macrophages, respectively (23). The role of CD169^+^ macrophages as efficient gatekeepers has been demonstrated in a large number of viral infections. Paradoxically, CD169^+^ macrophages can also support viral replication. Enforced viral replication within CD169^+^ macrophages endowed them with the distinct feature of being a source of viral antigen (24). We next sought to investigate which cells could potentially contribute to SARS-CoV-2 transport and replication in spleens and LNs, and results showed that SARS-CoV-2 NP also deposited in CD169^+^ macrophages. Previous work has showed that CD169^+^ macrophages are the first cell type in the spleen and LNs to bind to particulate antigens and pathogens and they function as a filter to remove foreign particles from the lymph fluid and blood (25), we therefore speculated that the CD169^+^ macrophages contribute to SARS-CoV-2 growth and spread in spleens and LNs *in vivo*.

We presently showed that the spleen and hilar LN tissues from COVID-19 autopsies manifested severe atrophy and lymphocyte reduction, lymphoid follicles are atrophied, decreased or absent, these results are similar to previously reported literatures (26, 27). Apoptosis-induced lymphocytopenia has been associated with a higher risk of infection and mortality in various clinical settings. For example, MERS-CoV could directly infect human primary T lymphocytes and induce T cell apoptosis (28). Here although the levels of FasL in spleens and hilar LNs from COVID-19 post-mortems are similar to those in tissues from age-matched controls, the expression of Fas is dramatically higher in secondary lymphoid organs from COVID-19 autopsies, suggesting that increased T cell apoptosis is one of the mechanisms responsible for systemic lymphocytopenia following SARS-CoV-2 infection. Dysregulated innate immune response with uncontrolled cytokine production is the most prominent clinical feature aggravating severe COVID-19 disease. Extravagant pro-inflammatory cytokines like IL-6 and IL-1β has been shown to mediate pyroptosis, a proinflammatory form of cell apoptosis (29). Here IHC confirmed that SARS-CoV-2 infected macrophages and DCs in spleens and LNs release high level of IL-6 and IL-1β, which can promote pro-apoptotic Fas upregulation (30). IL-10 is an inhibitory cytokine that not only induces T cell exhaustion, but also mediates cell apoptosis. Importantly, targeting tumors with IL-10 successfully prevents DCs-mediated CD8^+^ T Cell apoptosis (31). We here showed that 50.0% (9/18) perished COVID-19 cases have higher level of serum IL-10 than normal values, suggesting IL-10 might also involve in mediating lymphocytopenia. Additionally, CRP also has a major role in the apoptotic process. CRP stimulates the production of pro-apoptotic cytokines and inflammatory mediators, including IL-1β, TNF-α, and reactive oxygen species (ROS), *via* the activation of Fc-γ receptors (32). In the early stages of COVID-19, CRP levels were positively correlated with lung lesions and could reflect disease severity (33, 34). Here we showed that all of these patients had high serum concentrations of CRP (**Table 1**). These combined data suggested that both cytokine storm and CRP might be involved in inducing lymphocytopenia following SARS-CoV-2 infection.

Our previous work has demonstrated that serum concentrations of IL-6 are negatively correlated with circulating CD4^+^ and CD8^+^ T cell counts in COVID-19 patients (3), but little is known about the source of IL-6. We here shown that SARS-CoV-2 effectively triggered the transcription of *Il6* from infected MDMs and MoDCs, suggesting these viral infected immune cells are the major source of IL-6. SARS-CoV-2 infection has been shown to elevate mitochondrial ROS and glycolysis by stabilizing hypoxia inducible factor-1α (HIF-1α), which is necessary for viral replication and *Il6* transcription in monocytes (35), whether SARS-CoV-2 also hijacks HIF-1α/Glycolysis-dependent axis to promote viral replication and IL-6 secretion from DCs and macrophages need investigation. Tocilizumab, a humanized anti-IL-6 receptor antibody, has been approved for the treatment of COVID-19 patients, and results showed that it can alleviate clinical inflammatory response and efficiently enhance circulating lymphocyte counts (36). However, whether tocilizumab can restore lymphocyte counts in spleens and LNs of COVID-19 patients is an area requiring further investigation. Although data suggest that lymphocytopenia is probably caused by the translocation of lymphocytes from the peripheral blood to the lungs, the exact mechanism by which factors that induce lymphocyte translocation are unclear. IL-8, a potent neutrophil and T cell attractant and activator, plays a critical role in acute lung injury (37). Enhancement of IL-8 level was also reported in perished COVID-19 patients’ serum (38). We here also reported that the mean serum IL-8 concentration from these 18 fatal COVID-19 cases was 22.01 folds above the ULN (**Table 1**), further study is need to confirm whether IL-8 involves in mediating lymphocyte translocation.

We acknowledge that our study has several limitations. First, the number of patients in this study is relatively small, and the results should be validated in another prospective study. Second, there was no isolation of infectious SARS-CoV-2 virus and no quantification of viral genomes from the spleen or LN tissues due to the limits of the detection kits available now. Third, we found that SARS-CoV-2 infection can inhibit the transcription of IFN-I genes (*Ifna* and *Ifnb*), which is one of the major innate immune mechanisms against invading viral pathogens. However, the exact mechanisms underlying how SARS-CoV-2 disables these gene transcriptions are still unclear, although one study has shown that SARS-CoV-2 N protein antagonizes IFN-I signaling by suppressing phosphorylation and nuclear translocation of STAT1 and STAT2 (39). Fourth, we here only evaluated mRNA levels of *Il6 and Il1b from SARS-CoV-2 infected* MDMs and MoDCs by qRT-PCR, the amount of IL-6 and IL-1β in cultured supernatants which were inactivated by β-Propiolactone, however, is unsuccessfully detected by ELISA. Finally, we cannot exclude the possibility that some of the lymphocytopenia may be worsened due to the use of steroids during hospitalization, and further researches are required to determine the effects of corticosteroids on lymphocytes in the context of COVID-19.

In conclusion, we demonstrated that the SARS-CoV-2 virus can directly infect macrophages and DCs in human LNs and spleens, which leads to tissue damage and lymphocyte reduction through the promotion of IL-6 and IL-1β secretion. We also showed that CD169^+^ macrophages and CD11c^+^ DCs might play a central role in mediating SARS-CoV-2 translocation. Mechanistically, SASR-CoV-2 induces lymphocyte depletion in spleens and LNs by inducing Fas/FasL-dependent cell apoptosis, in addition to persistent viral antigen stimulation.

## Data Availability Statement

The original contributions presented in the study are included in the article/**Supplementary Material**. Further inquiries can be directed to the corresponding authors.

## Ethics Statement

The studies involving human participants were reviewed and approved by The Ethics Commission of the Jinyintan Hospital (KY-2020-15.01). The patients/participants provided their written informed consent to participate in this study.

## Author Contributions 

YW, and YC were involved in the final development of the project and manuscript preparation. QX, CHW, RC, ZF, HW and HY analyzed the data. BD and GW did H&E staining and immunohistochemistry. CSW evaluated H&E and immunohistochemistry results. QQ and YZ conducted Serum ELISA; LL, CT and RC provided autopsies. LDL performed viral infection *in vitro*. All authors contributed to the article and approved the submitted version.

## Funding

This work was supported by The National Key Research and Development Program of China (2016YFA0502204), and by the National Natural Science Foundation of China (NSFC; No. 81701562, 81701551, 81971478, and 81771691). The funding agencies did not participate in study design, data collection, data analysis, or manuscript writing.

## Conflict of Interest

The authors declare that the research was conducted in the absence of any commercial or financial relationships that could be construed as a potential conflict of interest.
