# Supplementary material for: SARS-CoV-2 Induces Lymphocytopenia by Promoting Inflammation and Decimates Secondary Lymphoid Organs
Source: Front Immunol. 2021 Apr 28;12:661052. doi: 10.3389/fimmu.2021.661052 (PMC8113960; doi:10.3389/fimmu.2021.661052)
Supplement: Supplementary file 1 [file Image_1.pdf]

Figure S1 Representative morphology of lung tissues stained by H&E.

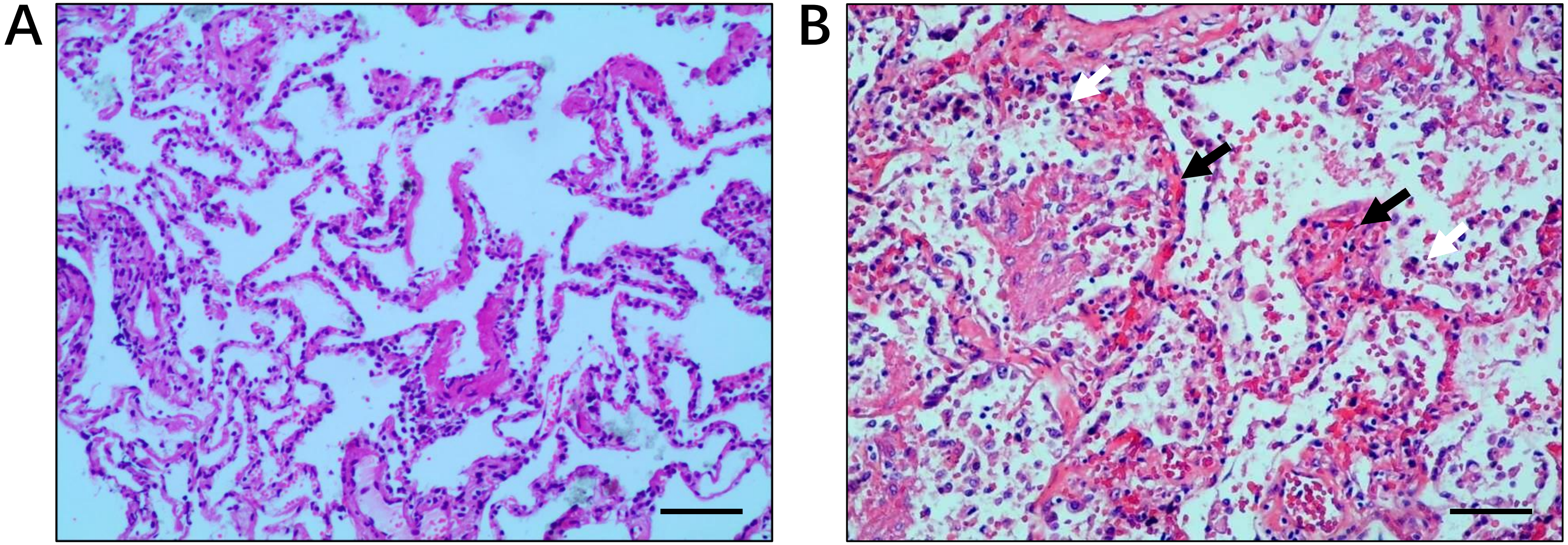

Lung sections from **(A)** one representative trauma victim, **(B)** one COVID-19 patient undergoing postmortem examination (case #2) were stained by H&E staining. Black arrow indicated thickening alveolar wall, white arrow head indicated infiltrated inflammatory cells. Scale bar= 100 μM.

Figure S2 Compared the histopathology of spleen and LN tissues from indicated patients.

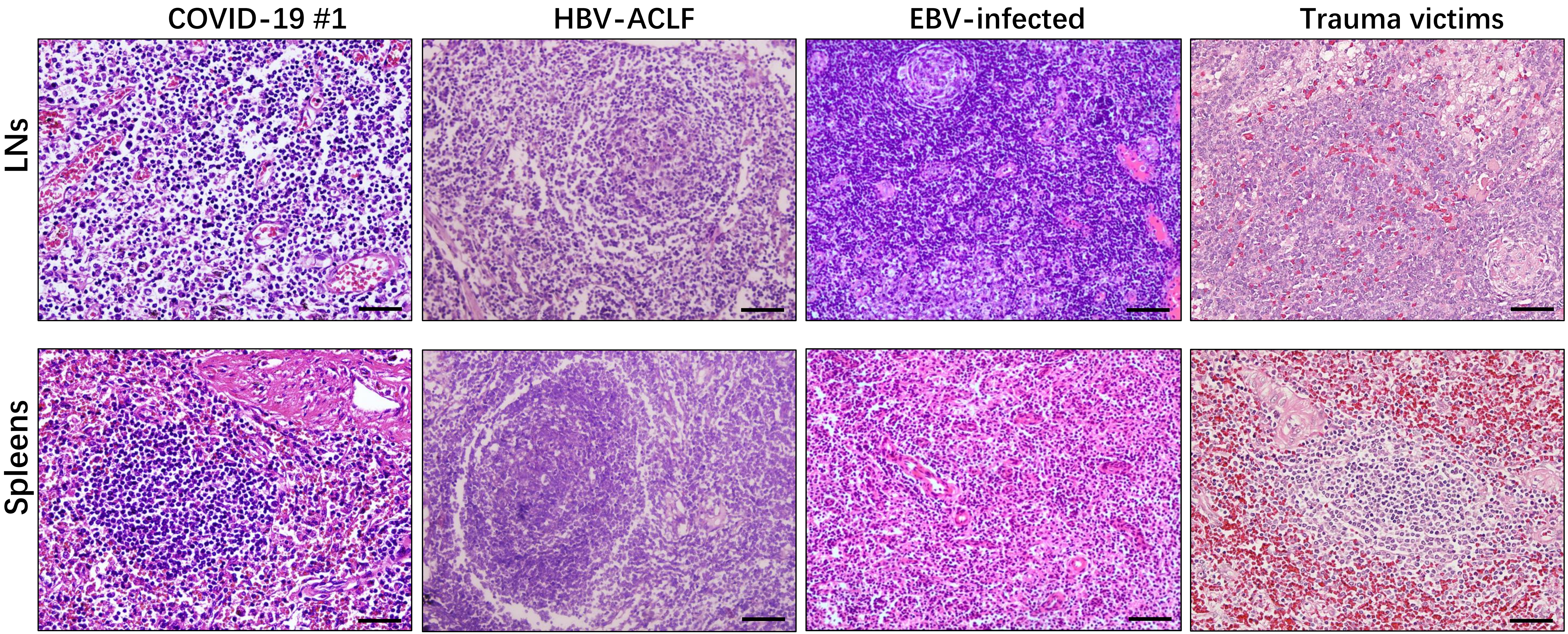

Sections from COVID-19 (case #1) manifested severe cell apoptosis and tissue damage, whereas, sections from HBV-ACLF patients and trauma victims showed normal morphology, and sections from EBV-infected patients manifested with smaller lymphoid follicles, T zone proliferation and enhancement of immunoblastic cells. Scale bar= 100 μM.

Figure S3 The expression of FasL in the spleens and LNs was detected by IHC.

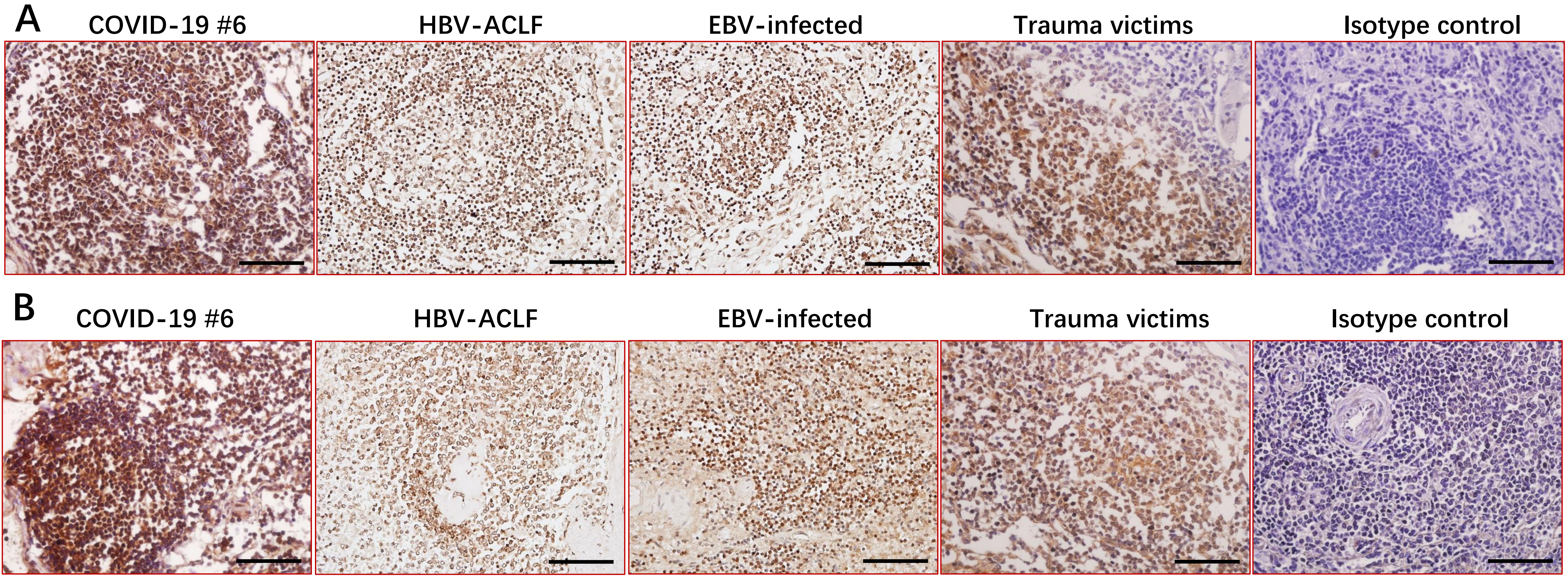

The expression of FasL in sections from LNs (A) and spleens (B) of indicated patients were detected by IHC. Brown color indicates positive cells, scale bar= 100 μM.

Figure S4 SARS-CoV-2 RNA was undetected in the spleens from trauma victim and mock infected human macrophages and DCs.

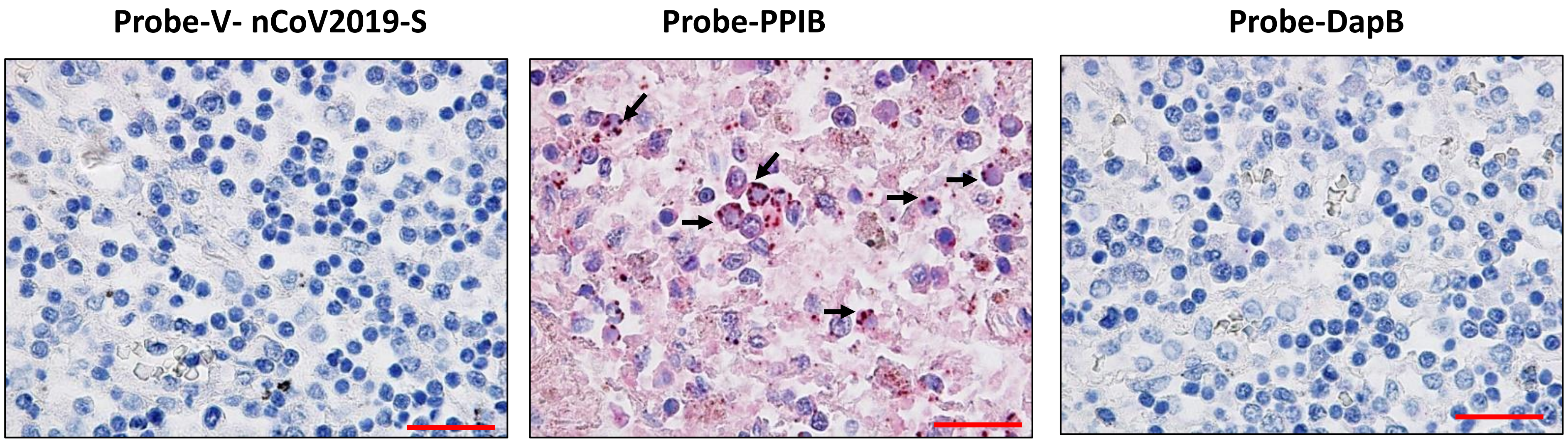

SARS-CoV-2 RNA in spleen sections from trauma victim was detected by ISH, results showed negative reactivity for Probe-V- nCoV2019-S**.** Here probes to the housekeeping gene PPIB were used as positive controls. Arrowed indicated positive cells, scale bar= 50 μM.

Figure S5 The expression of SARS-CoV-2 NP antigen in the spleens and LNs from indicated patients.

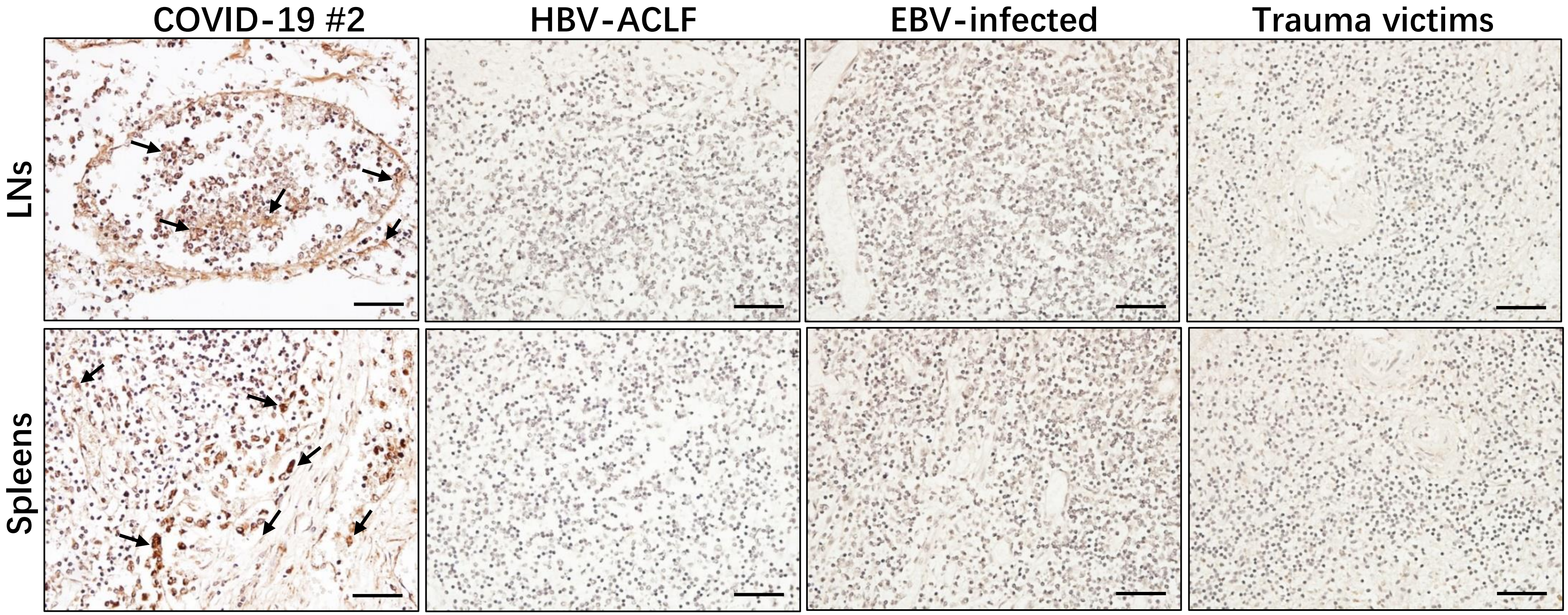

The expression of SARS-CoV-2 NP antigen in the spleen and LN sections from indicated patients and one COVID-19 patient undergoing postmortem examination (case #2) were detected by IHC. Arrowed indicated positive cells, scale bar= 100 μM.

Table S1 Demographics and baseline characteristics of perished patients with COVID-19.

Cases	Sex	Age	Underlying diseases	Fever	Cough	Dyspnea	Fatigue	Myalgia	Expectoration	Respiratory failure	Secondary infection	Acute cardiac injury	Acute kidney injury	Acute liver injury	Corticosteroid	CPAP	Hospital days
#1	Male	62	No	Yes	Ye	Yes	Yes	Yes	No	No	Yes	No	No	No	Yes	Yes	50
#2	Male	53	No	Yes	Ye	Yes	No	No	No	No	No	No	No	No	Yes	Yes	41
#3	Male	73	Hypertension	No	Ye	Yes	Yes	No	No	No	No	No	No	No	Yes	Yes	46
#4	Male	67	No	Yes	No	Yes	Yes	Yes	No	No	No	No	No	No	Yes	Yes	28
#5	Male	81	No	Yes	No	Yes	Yes	No	No	Yes	Yes	No	Yes	Yes	Yes	No	44
#6	Male	75	Hypertension/Diabete	Yes	Ye	Yes	No	No	No	No	No	Yes	Yes	No	Yes	Yes	49
#7	Male	82	Hypertension	Yes	No	No	Yes	No	No	No	No	No	No	No	Yes	Yes	57
#8	Male	87	Diabete	No	Ye	Yes	Yes	No	Yes	Yes	No	Yes	Yes	No	Yes	Yes	44
#9	Male	77	Diabete	Yes	No	Yes	No	No	No	No	No	Yes	No	No	Yes	Yes	64
#10	Male	89	No	Yes	Ye	No	Yes	No	Yes	No	Yes	No	No	No	Yes	Yes	18
#11	Female	89	No	Yes	No	Yes	No	No	No	No	No	Yes	Yes	No	Yes	Yes	17
#12	Male	83	Hypertension/Diabete	No	Ye	Yes	No	No	No	Yes	Yes	Yes	Yes	No	Yes	No	44
#13	Female	58	Hypertension	Yes	Ye	Yes	No	No	No	No	No	No	No	No	Yes	Yes	67
#14	Female	84	Hypertension	Yes	Ye	Yes	Yes	Yes	No	Yes	No	No	No	No	Yes	Yes	47
#15	Male	70	No	No	Ye	Yes	Yes	No	No	Yes	No	No	No	No	Yes	Yes	38
#16	Male	82	Hypertension	Yes	No	Yes	No	No	No	No	No	Yes	No	No	Yes	Yes	18
#17	Male	70	Hypertension	Yes	No	Yes	No	No	No	Yes	No	No	No	No	Yes	Yes	27
#18	Female	65	Hypertension	Yes	Yes	Yes	Yes	No	No	Yes	No	No	No	No	Yes	Yes	49

CPAP: Continuous Postive Airway Pressure

Table S2 Laboratory findings of perished patients with COVID-19.

Cases	Fibrinogen （g/L)	ALT (IU/L)	AST (IU/L)	TP (g/L)	ALB (g/L)	GLB (g/L)	TBIL (umol/L)	IBIL (umol/L)	TBA (umol/L)	GLU (umol/L)	Cre (umol/L)	UA (umol/L)	ALP (IU/L)	GGT (IU/L)	CK-MB (IU/L)	CysC (mg/L)	K (mmol/L)	Na (mmol/L)	CL (mmol/L)	Ca (mmol/L)	P (mmol/L)	Mg (mmol/L)
#1	3.92	21.1	37.3	61.3	41.6	19.7	554.5	178.51	43.6	3.94	63.8	144	162.4	25	18.5	1.13	3.95	155.7	101.2	2.61	0.01	0.85
#2	2.83	320.5	82.2	58.6	36.7	21.9	13	4.85	5	7.81	63.5	130	57.1	68.7	7.6	1.76	5.09	146.5	108.6	2.1	1.13	0.93
#3	3.48	17.1	17.3	56.2	34.7	21.5	21	11.89	2.9	6.75	56.3	294	65.5	34	29.5	1.8	4.14	153.2	114.8	2.08	0.5	0.85
#4	3.77	77.8	102	63.3	35.5	27.8	7.5	3.43	10.1	8.51	64.7	104	102.9	80.5	14	1.64	4.67	142.6	107.2	2.17	0.63	1.11
#5	1.04	14.2	23.3	56	30.9	25.1	35.5	12.7	17.5	5.2	56.3	331	86.2	41.4	26.9	1.6	3.58	157.3	117.1	2.19	0.64	1.03
#6	3.93	8.6	16.7	54.1	26.7	27.4	5.8	3.5	0	8.92	232.9	801	51.6	26.1	72	2.54	4.64	165.1	130.1	2	1.66	1.52
#7	1.84	27	54.5	46.3	27.6	18.7	32.6	10.72	37.2	7.51	73.8	224	254.6	27.6	12.4	1.49	4.5	136.6	106.2	1.98	0.92	0.89
#8	1.57	16.6	26.9	49.8	27.1	22.7	90.3	51	32.3	5.7	89.9	275	161.2	42.6	17.3	2.67	3.88	155.6	112.1	2.34	0.01	0.88
#9	1.17	43.7	86.2	40.4	27.1	13.3	46.1	2.33	84.3	11.24	58.9	269	272	32.5	23.8	2.28	5.9	140.5	98.9	2.22	1.54	1.08
#10	2.11	9.70	21.90	47.3	27.3	20.0	15.6	4.51	5.1	6.40	144.4	523	134.30	20.10	12.80	1.88	3.15	153.8	107.70	3.48	1.90	1.10
#11	2.96	7.80	13.10	56.8	27.6	29.2	5.8	2.89	10.4	6.27	76.0	258	29.40	6.30	16.80	3.06	4.68	136.0	104.80	1.91	0.80	0.86
#12	1.48	43.50	94.80	43.3	29.0	14.3	132.2	35.60	42.4	7.73	49.1	35	85.80	80.40	47.80	0.86	4.14	140.6	108.40	2.18	0.33	0.91
#13	2.46	29.30	39.50	78.5	50.1	28.4	24.5	6.60	8.1	7.86	30.8	48	134.50	124.10	37.40	0.64	4.68	140.2	102.20	2.51	0.31	1.10
#14	2.40	20.80	32.00	58.6	37.1	21.5	12.3	4.47	8.0	10.22	41.1	93	118.50	20.40	27.10	0.64	4.60	146.1	106.80	2.20	0.73	1.05
#15	2.48	>1000	>800	40.3	25.2	15.1	45.0	10.79	42.3	3.76	40.2	146	214.90	54.50	233.50	0.61	4.29	148.1	104.30	1.84	0.65	1.00
#16	1.38	64.00	77.30	52.6	25.7	26.9	17.5	7.24	5.1	6.78	77.6	192	108.00	57.00	18.80	2.04	4.01	137.8	103.90	1.91	0.57	0.78
#17	3.50	43.80	25.70	54.1	25.8	28.3	10.1	4.25	4.3	8.29	54.2	202	107.80	104.40	18.70	1.15	4.12	143.9	106.70	1.86	0.81	1.02
#18	4.20	13.60	17.00	50.7	30.8	19.9	3.3	1.00	9.3	10.70	154.4	306	77.50	20.10	11.80	2.21	4.35	152.0	111.60	2.08	1.53	0.74
Mean	2.60	60.73	49.60	54.00	31.99	22.01	89.59	30.99	25.88	7.29	84.46	285.78	134.83	42.04	24.67	1.88	4.48	150.34	110.69	2.19	0.78	1.02
Normal range	2-4 g/L	9-50	9-60	65-85	40-55	20-40	0-26	0-14	0-10	3.9-6.11	57-97	202-416	45-125	10-60	0-24	0.63-1.25	3.5-5.3	137-147	99-110	2.11-2.52	0.85-1.51	0.75-1.02

ALT:Alanine aminotransferase
AST: Aspartate aminotransferase
TP:Total protein
ALB: Albumin
GLB: Globulin
TBIL: Total bilirubin
IBIL: Indirect bilirubin
TBA: Total biliary acid
GLU: Glucose
Cre: Creatinine
UA: Uric acid
ALP: Alkaline phosphatase
GGT: Gamma glutamyltranspeptidase
CK-MB: Creatine kinase-MB
CysC: Cystatin C

Table S3 The primer Sequences for qPCR in this research.

**Genes**	**Primer Sequence (forward) 5'- 3'**	**Primer Sequence (reverse) 5'- 3'**
IL-6	ACTCACCTCTTCAGAACGAATTG	CCATCTTTGGAAGGTTCAGGTTG
IL-1β	GGACAAGCTGAGGAAGATGC	TCGTTATCCCATGTGTCGAA
TNF-α	TCCTTCAGACACCCTCAACC	AGGCCCCAGTTTGAATTCTT
IFN-α	ACCCACAGCCTGGATAACAG	ACTGGTTGCCATCAAACTCC
IFN-β	TGGGAGGATTCTGCATTACC	CAGCATCTGCTGGTTGAAGA
IFN-γ	TCGGTAACTGACTTGAATGTCCA	TCGCTTCCCTGTTTTAGCTGC
IL-10	GACTTTAAGGGTTACCTGGGTTG	TCACATGCGCCTTGATGTCTG
IL-18	TCTTCATTGACCAAGGAAATCGG	TCCGGGGTGCATTATCTCTAC
IL-8	TAGCAAAATTGAGGCCAAGG	AAACCAAGGCACAGTGGAAC
ACE2	CAAGAGCAAACGGTTGAACAC	CCAGAGCCTCTCATTGTAGTCT
HPRT	CCTGGCGTCGTGATTAGTGAT	AGACGTTCAGTCCTGTCCATAA
SARS-CoV2-NP	CATTGGCATGGAAGTCACAC	TCTGCGGTAAGGCTTGAGTT
SARS-CoV2-S	CTTCCCTCAGTCAGCACCTC	AACCAGTGTGTGCCATTTGA
